# Supplementary material for: Rapid Support for Older Adults during the Initial Stages of the COVID-19 Pandemic: Results from a Geriatric Psychiatry Helpline
Source: Geriatrics (Basel). 2021 Mar 22;6(1):30. doi: 10.3390/geriatrics6010030 (PMC8006039; doi:10.3390/geriatrics6010030)
Supplement: Supplementary file 1 [file geriatrics-06-00030-s001.pdf]

Supplementary Table 1. Skewness and Kurtorsis for mental health variables of depression and anxiety. SE Standard Error. Both variables were sufficiently normally distributed.

		Skewness		Kurtosis	
	**N**	**Value**	***SE***	**Value**	***SE***
Anxiety	35	-0.058	0.398	-1.271	0.778
Depression	44	0.293	0.357	-0.908	0.702

SD= Standard deviation. SE= Standard error

Supplementary Table 2. Table depicting the different features of help provided and subsequent appointments scheduled by us for psychiatric or psychotherapeutic counseling or support by our social worker. Total N represents the number of callers which participated in the survey. N responded= the number of callers which were willing to answer the question. N represents the number of callers which affirmed the specific information and features asked. The percentage was calculated as (N/N responded)*100.

	**N or M (SD)**	**(%)**
**Result of the helpline call (N responded= 51)**		
Providing information of Covid-19	30	58.8
Practical help/information for daily living	1	1.9
Providing social contacts	1	1.9
Providing help for somatic diseases/issues	7	13.7
Recommendation for psychiatric counseling	15	29.4
Providing relieving psychological support	29	56.9
Providing contact to welfare work	1	1.9
**Subsequent treatments (N responded= 51)**		
No subsequent treatments	38	74.5
Subsequent treatments required	10	19.6
Subsequent psychiatric treatment provided	3	5.9
Subsequent psychotherapy provided	7	13.7
Subsequent welfare work provided	1	1.9
